# Critical Thinking: A Model of Intelligence for Solving Real-World Problems

**DOI:** 10.3390/jintelligence9020022

**Published:** 2021-04-07

**Authors:** Diane F. Halpern, Dana S. Dunn

**Affiliations:** 1 Department of Psychology, Claremont McKenna College, Emerita, Altadena, CA 91001, USA; 2 Department of Psychology, Moravian College, Bethlehem, PA 18018, USA; dunn@moravian.edu

**Keywords:** critical thinking, intelligence, real-world problems

## Abstract

Most theories of intelligence do not directly address the question of whether people with high intelligence can successfully solve real world problems. A high IQ is correlated with many important outcomes (e.g., academic prominence, reduced crime), but it does not protect against cognitive biases, partisan thinking, reactance, or confirmation bias, among others. There are several newer theories that directly address the question about solving real-world problems. Prominent among them is Sternberg’s adaptive intelligence with “adaptation to the environment” as the central premise, a construct that does not exist on standardized IQ tests. Similarly, some scholars argue that standardized tests of intelligence are not measures of rational thought—the sort of skill/ability that would be needed to address complex real-world problems. Other investigators advocate for critical thinking as a model of intelligence specifically designed for addressing real-world problems. Yes, intelligence (i.e., critical thinking) can be enhanced and used for solving a real-world problem such as COVID-19, which we use as an example of contemporary problems that need a new approach.

## 1. Introduction

The editors of this Special Issue asked authors to respond to a deceptively simple statement: *“How Intelligence Can Be a Solution to Consequential World Problems.”* This statement holds many complexities, including how intelligence is defined and which theories are designed to address real-world problems.

## 2. The Problem with Using Standardized IQ Measures for Real-World Problems

For the most part, we identify high intelligence as having a high score on a standardized test of intelligence. Like any test score, IQ can only reflect what is on the given test. Most contemporary standardized measures of intelligence include vocabulary, working memory, spatial skills, analogies, processing speed, and puzzle-like elements (e.g., Wechsler Adult Intelligence Scale Fourth Edition; see (Drozdick et al. 2012)). Measures of IQ correlate with many important outcomes, including academic performance (Kretzschmar et al. 2016), job-related skills (Hunter and Schmidt 1996), reduced likelihood of criminal behavior (Burhan et al. 2014), and for those with exceptionally high IQs, obtaining a doctorate and publishing scholarly articles (McCabe et al. 2020). Gottfredson (1997, p. 81) summarized these effects when she said the “predictive validity of g is ubiquitous.” More recent research using longitudinal data, found that general mental abilities and specific abilities are good predictors of several work variables including job prestige, and income (Lang and Kell 2020). Although assessments of IQ are useful in many contexts, having a high IQ does not protect against falling for common cognitive fallacies (e.g., blind spot bias, reactance, anecdotal reasoning), relying on biased and blatantly one-sided information sources, failing to consider information that does not conform to one’s preferred view of reality (confirmation bias), resisting pressure to think and act in a certain way, among others. This point was clearly articulated by Stanovich (2009, p. 3) when he stated that,” IQ tests measure only a small set of the thinking abilities that people need.”

## 3. Which Theories of Intelligence Are Relevant to the Question?

Most theories of intelligence do not directly address the question of whether people with high intelligence can successfully solve real world problems. For example, Grossmann et al. (2013) cite many studies in which IQ scores have not predicted well-being, including life satisfaction and longevity. Using a stratified random sample of Americans, these investigators found that wise reasoning is associated with life satisfaction, and that “there was no association between intelligence and well-being” (p. 944). (critical thinking [CT] is often referred to as “wise reasoning” or “rational thinking,”). Similar results were reported by Wirthwein and Rost (2011) who compared life satisfaction in several domains for gifted adults and adults of average intelligence. There were no differences in any of the measures of subjective well-being, except for leisure, which was significantly lower for the gifted adults. Additional research in a series of experiments by Stanovich and West (2008) found that participants with high cognitive ability were as likely as others to endorse positions that are consistent with their biases, and they were equally likely to prefer one-sided arguments over those that provided a balanced argument. There are several newer theories that directly address the question about solving real-world problems. Prominent among them is Sternberg’s adaptive intelligence with “adaptation to the environment” as the central premise, a construct that does not exist on standardized IQ tests (e.g., Sternberg 2019). Similarly, Stanovich and West (2014) argue that standardized tests of intelligence are not measures of rational thought—the sort of skill/ability that would be needed to address complex real-world problems. Halpern and Butler (2020) advocate for CT as a useful model of intelligence for addressing real-world problems because it was designed for this purpose. Although there is much overlap among these more recent theories, often using different terms for similar concepts, we use Halpern and Butler’s conceptualization to make our point: Yes, intelligence (i.e., CT) can be enhanced and used for solving a real-world problem like COVID-19.

## 4. Critical Thinking as an Applied Model for Intelligence

One definition of intelligence that directly addresses the question about intelligence and real-world problem solving comes from Nickerson (2020, p. 205): “the ability to learn, to reason well, to solve novel problems, and to deal effectively with novel problems—often unpredictable—that confront one in daily life.” Using this definition, the question of whether intelligent thinking can solve a world problem like the novel coronavirus is a resounding “yes” because solutions to real-world novel problems are part of his definition. This is a popular idea in the general public. For example, over 1000 business managers and hiring executives said that they want employees who can think critically based on the belief that CT skills will help them solve work-related problems (Hart Research Associates 2018).

We define CT as the use of those cognitive skills or strategies that increase the probability of a desirable outcome. It is used to describe thinking that is purposeful, reasoned, and goal directed--the kind of thinking involved in solving problems, formulating inferences, calculating likelihoods, and making decisions, when the thinker is using skills that are thoughtful and effective for the particular context and type of thinking task. International surveys conducted by the OECD (2019, p. 16) established “key information-processing competencies” that are “highly transferable, in that they are relevant to many social contexts and work situations; and ‘learnable’ and therefore subject to the influence of policy.” One of these skills is problem solving, which is one subset of CT skills.

The CT model of intelligence is comprised of two components: (1) understanding information at a deep, meaningful level and (2) appropriate use of CT skills. The underlying idea is that CT skills can be identified, taught, and learned, and when they are recognized and applied in novel settings, the individual is demonstrating intelligent thought. CT skills include judging the credibility of an information source, making cost–benefit calculations, recognizing regression to the mean, understanding the limits of extrapolation, muting reactance responses, using analogical reasoning, rating the strength of reasons that support and fail to support a conclusion, and recognizing hindsight bias or confirmation bias, among others. Critical thinkers use these skills appropriately, without prompting, and usually with conscious intent in a variety of settings.

One of the key concepts in this model is that CT skills transfer in appropriate situations. Thus, assessments using situational judgments are needed to assess whether particular skills have transferred to a novel situation where it is appropriate. In an assessment created by the first author (Halpern 2018), short paragraphs provide information about 20 different everyday scenarios (e.g., A speaker at the meeting of your local school board reported that when drug use rises, grades decline; so schools need to enforce a “war on drugs” to improve student grades); participants provide two response formats for every scenario: (a) constructed responses where they respond with short written responses, followed by (b) forced choice responses (e.g., multiple choice, rating or ranking of alternatives) for the same situations.

There is a large and growing empirical literature to support the assertion that CT skills can be learned and will transfer (when taught for transfer). See for example, Holmes et al. (2015), who wrote in the prestigious *Proceedings of the National Academy of Sciences*, that there was “significant and sustained improvement in students’ critical thinking behavior” (p. 11,199) for students who received CT instruction. Abrami et al. (2015, para. 1) concluded from a meta-analysis that “there are effective strategies for teaching CT skills, both generic and content specific, and CT dispositions, at all educational levels and across all disciplinary areas.” Abrami et al. (2008, para. 1), included 341 effect sizes in a meta-analysis. They wrote: “findings make it clear that improvement in students’ CT skills and dispositions cannot be a matter of implicit expectation.” A strong test of whether CT skills can be used for real-word problems comes from research by Butler et al. (2017). Community adults and college students (N = 244) completed several scales including an assessment of CT, an intelligence test, and an inventory of real-life events. Both CT scores and intelligence scores predicted individual outcomes on the inventory of real-life events, but CT was a stronger predictor.

Heijltjes et al. (2015, p. 487) randomly assigned participants to either a CT instruction group or one of six other control conditions. They found that “only participants assigned to CT instruction improved their reasoning skills.” Similarly, when Halpern et al. (2012) used random assignment of participants to either a learning group where they were taught scientific reasoning skills using a game format or a control condition (which also used computerized learning and was similar in length), participants in the scientific skills learning group showed higher proportional learning gains than students who did not play the game. As the body of additional supportive research is too large to report here, interested readers can find additional lists of CT skills and support for the assertion that these skills can be learned and will transfer in Halpern and Dunn (Forthcoming). There is a clear need for more high-quality research on the application and transfer of CT and its relationship to IQ.

## 5. Pandemics: COVID-19 as a Consequential Real-World Problem

A pandemic occurs when a disease runs rampant over an entire country or even the world. Pandemics have occurred throughout history: At the time of writing this article, COVID-19 is a world-wide pandemic whose actual death rate is unknown but estimated with projections of several million over the course of 2021 and beyond (Mega 2020). Although vaccines are available, it will take some time to inoculate most or much of the world’s population. Since March 2020, national and international health agencies have created a list of actions that can slow and hopefully stop the spread of COVID (e.g., wearing face masks, practicing social distancing, avoiding group gatherings), yet many people in the United States and other countries have resisted their advice.

Could instruction in CT encourage more people to accept and comply with simple life-saving measures? There are many possible reasons to believe that by increasing citizens’ CT abilities, this problematic trend can be reversed for, at least, some unknown percentage of the population. We recognize the long history of social and cognitive research showing that changing attitudes and behaviors is difficult, and it would be unrealistic to expect that individuals with extreme beliefs supported by their social group and consistent with their political ideologies are likely to change. For example, an Iranian cleric and an orthodox rabbi both claimed (separately) that the COVID-19 vaccine can make people gay (Marr 2021). These unfounded opinions are based on deeply held prejudicial beliefs that we expect to be resistant to CT. We are targeting those individuals who beliefs are less extreme and may be based on reasonable reservations, such as concern about the hasty development of the vaccine and the lack of long-term data on its effects. There should be some unknown proportion of individuals who can change their COVID-19-related beliefs and actions with appropriate instruction in CT. CT can be a (partial) antidote for the chaos of the modern world with armies of bots creating content on social media, political and other forces deliberately attempting to confuse issues, and almost all media labeled “fake news” by social influencers (i.e., people with followers that sometimes run to millions on various social media). Here, are some CT skills that could be helpful in getting more people to think more critically about pandemic-related issues.

### Reasoning by Analogy and Judging the Credibility of the Source of Information

Early communications about the ability of masks to prevent the spread of COVID from national health agencies were not consistent. In many regions of the world, the benefits of wearing masks incited prolonged and acrimonious debates (Tang 2020). However, after the initial confusion, virtually all of the global and national health organizations (e.g., WHO, National Health Service in the U. K., U. S. Centers for Disease Control and Prevention) endorse masks as a way to slow the spread of COVID (Cheng et al. 2020; Chu et al. 2020). However, as we know, some people do not trust governmental agencies and often cite the conflicting information that was originally given as a reason for not wearing a mask. There are varied reasons for refusing to wear a mask, but the one most often cited is that it is against civil liberties (Smith 2020). Reasoning by analogy is an appropriate CT skill for evaluating this belief (and a key skill in legal thinking). It might be useful to cite some of the many laws that already regulate our behavior such as, requiring health inspections for restaurants, setting speed limits, mandating seat belts when riding in a car, and establishing the age at which someone can consume alcohol. Individuals would be asked to consider how the mandate to wear a mask compares to these and other regulatory laws.

Another reason why some people resist the measures suggested by virtually every health agency concerns questions about whom to believe. Could training in CT change the beliefs and actions of even a small percentage of those opposed to wearing masks? Such training would include considering the following questions with practice across a wide domain of knowledge: (a) Does the source have sufficient expertise? (b) Is the expertise recent and relevant? (c) Is there a potential for gain by the information source, such as financial gain? (d) What would the ideal information source be and how close is the current source to the ideal? (e) Does the information source offer evidence that what they are recommending is likely to be correct? (f) Have you traced URLs to determine if the information in front of you really came from the alleged source?, etc. Of course, not everyone will respond in the same way to each question, so there is little likelihood that we would all think alike, but these questions provide a framework for evaluating credibility. Donovan et al. (2015) were successful using a similar approach to improve dynamic decision-making by asking participants to reflect on questions that relate to the decision. Imagine the effect of rigorous large-scale education in CT from elementary through secondary schools, as well as at the university-level. As stated above, empirical evidence has shown that people can become better thinkers with appropriate instruction in CT. With training, could we encourage some portion of the population to become more astute at judging the credibility of a source of information? It is an experiment worth trying.

## 6. Making Cost—Benefit Assessments for Actions That Would Slow the Spread of COVID-19

Historical records show that refusal to wear a mask during a pandemic is not a new reaction. The epidemic of 1918 also included mandates to wear masks, which drew public backlash. Then, as now, many people refused, even when they were told that it was a symbol of “wartime patriotism” because the 1918 pandemic occurred during World War I (Lovelace 2020). CT instruction would include instruction in why and how to compute cost–benefit analyses. Estimates of “lives saved” by wearing a mask can be made meaningful with graphical displays that allow more people to understand large numbers. Gigerenzer (2020) found that people can understand risk ratios in medicine when the numbers are presented as frequencies instead of probabilities. If this information were used when presenting the likelihood of illness and death from COVID-19, could we increase the numbers of people who understand the severity of this disease? Small scale studies by Gigerenzer have shown that it is possible.

### Analyzing Arguments to Determine Degree of Support for a Conclusion

The process of analyzing arguments requires that individuals rate the strength of support for and against a conclusion. By engaging in this practice, they must consider evidence and reasoning that may run counter to a preferred outcome. Kozyreva et al. (2020) call the deliberate failure to consider both supporting and conflicting data “deliberate ignorance”—avoiding or failing to consider information that could be useful in decision-making because it may collide with an existing belief. When applied to COVID-19, people would have to decide if the evidence for and against wearing a face mask is a reasonable way to stop the spread of this disease, and if they conclude that it is not, what are the costs and benefits of not wearing masks at a time when governmental health organizations are making them mandatory in public spaces? Again, we wonder if rigorous and systematic instruction in argument analysis would result in more positive attitudes and behaviors that relate to wearing a mask or other real-world problems. We believe that it is an experiment worth doing.

## 7. Conclusions

We believe that teaching CT is a worthwhile approach for educating the general public in order to improve reasoning and motivate actions to address, avert, or ameliorate real-world problems like the COVID-19 pandemic. Evidence suggests that CT can guide intelligent responses to societal and global problems. We are NOT claiming that CT skills will be a universal solution for the many real-world problems that we confront in contemporary society, or that everyone will substitute CT for other decision-making practices, but we do believe that systematic education in CT can help many people become better thinkers, and we believe that this is an important step toward creating a society that values and practices routine CT. The challenges are great, but the tools to tackle them are available, if we are willing to use them.
